# Steroid insensitive fixed airflow obstruction is not related to airway inflammation in older non-smokers with asthma

**DOI:** 10.1186/s12931-018-0880-2

**Published:** 2018-09-17

**Authors:** K. O. Tonga, G. G. King, C. S. Farah, C. Thamrin, F. S. Tang, J. Santos, P. Sharma, D. G. Chapman, B. G. Oliver

**Affiliations:** 1 Woolcock Institute of Medical Research, The University of Sydney, Glebe New South Wales, Sydney, NSW 2037 Australia; 2 Department of Respiratory Medicine, Royal North Shore Hospital, St Leonards, NSW Australia; 3 Department of Respiratory Medicine, Concord Hospital, Concord, NSW Australia; 4 The University of Sydney, School of Medicine, Faculty of Medicine and Health, Sydney, NSW Australia; 5 Macquarie University, Faculty of Medicine and Health Sciences, North Ryde, NSW Australia; 6 Discipline of Medical Sciences, University of Technology Sydney, Broadway, Sydney, NSW Australia; 7 School of Life Sciences, University of Technology Sydney, Ultimo, NSW Australia; 8 CIRIMAT, University of Toulouse, Toulouse, France; 9 St Vincent’s Clinical School, University of New South Wales Medicine, University of New South Wales, Darlinghurst, NSW Australia

**Keywords:** Fixed airflow obstruction, Asthma, Reduced lung elastic recoil, Airway inflammation

## Abstract

There is limited evidence linking airway inflammation and lung function impairment in older non-smoking asthmatics with fixed airflow obstruction (FAO), which can develop despite treatment with inhaled corticosteroids (ICS). We assessed lung function (spirometry, forced oscillation technique (FOT)), lung elastic recoil and airway inflammation using bronchoalveolar lavage (BAL) in non-smoking adult asthmatics with FAO, following 2 months treatment with high-dose ICS/long-acting beta-agonist. Subjects demonstrated moderate FAO, abnormal FOT indices and loss of lung elastic recoil. This cross-sectional study showed a lack of a relationship between BAL neutrophils, eosinophils, inflammatory cytokines and lung function impairment. Other inflammatory pathways or the effect of inflammation on lung function over time may explain FAO development.

Irreversible or fixed airflow obstruction (FAO) can develop in long-standing asthma despite no or minimal smoking history and is associated with moderate to severe disease [1, 2]. The mechanisms of FAO in asthma are poorly understood; therefore prevention and treatment remain a challenge. Inhaled and/or oral corticosteroids improve lung function and reduce exacerbations, yet may not necessarily prevent FAO from occurring [3, 4]. Asthma severity [1, 5] and FAO development may be attributed to corticosteroid resistance or insensitivity resulting in persistent airway inflammation [6] and structural airway changes. Both eosinophilic and neutrophilic inflammation may be associated with lung function impairment and FAO in asthma, however evidence is limited and contradictory [4, 7].

In this prospective study, we investigated whether lung function impairment in older non-smokers with long-standing asthma and FAO is associated with the airway inflammation which remains after treatment with maximal dose inhaled corticosteroid (ICS). We hypothesized that the degree of lung function abnormalities would positively correlate with persistent airway inflammation in patients with asthma and FAO, thereby providing a potential mechanism for the development of FAO and its apparent steroid insensitivity.

Patients were > 40 years old, non-smokers or had a negligible smoking history with a respiratory physician diagnosis of asthma. All patients were treated with a standardized maximal dose of ICS/long-acting beta-agonist (ICS/LABA) using fluticasone/eformoterol 250 μg/10 μg metered dose inhaler via a holding chamber, two puffs twice daily, if not already taking this treatment. A baseline test skin prick test to common allergens was performed to assess atopic status. During enrolment and after 2 months of treatment, patients completed a symptom questionnaire (Asthma Control Questionnaire, ACQ-5) and performed pre-bronchodilator lung function measurements. Measurements included spirometry and the forced oscillation technique (FOT) to derive airway resistance (R_5_) and reactance (X_5_) at 5 Hz. After 2 months of treatment an oesphageal balloon was used to derive the pressure-volume (P-V) curve to assess the elastic recoil properties of the lung via the indices K, reflecting lung compliance, and B/A, reflecting lung elastic recoil [8]. FAO was assessed following 1 month of treatment and was defined as a < 200 ml and < 12% change in spirometry post-bronchodilator (400mcg inhaled salbutamol). ICS/LABA medication was withheld for at least 24 h and short acting beta-agonist medication for at least 6 h prior to testing.

Following 2 months of ICS/LABA treatment and within a week of the lung function measurements, patients then underwent bronchoscopy with bronchoalveolar lavage (BAL) from the right middle lobe [9]. Neutrophil and eosinophil counts were obtained from BAL samples as previously described [10]. Cytokines including IL-1b, IL-4, IL-6, IL-10, IL-17A, IL-17F, IL-21, IL-22, IL-23, IL-25, IL-31, IL-33, IFN-γ, scD40L and TNF-α were measured in BAL supernatant using a multiplex immunoassay (Bio-Rad® Bio-Plex Multiplex Immunoassay). Univariate correlations (Spearman rank test) between lung function indices (using z-scores) and BAL samples (using raw values) after 2 months of treatment were assessed.

Nineteen patients were recruited (11 male; mean ± SD age 63 ± 9 years, asthma duration 38 ± 22 years, height 1.69 ± 0.10 m, body mass index 28.4 ± 5.8 kg/metre^2^); 18 completed the study. Five patients were ex-smokers with 2.2 ± 2.5 pack-years smoking history and 14/19 patients were atopic. Patients were symptomatic (ACQ-5 1.03 ± 0.92) despite taking regular asthma medications prior to enrolment (ICS 18/19; with LABA 18/19; ICS/LABA/long-acting muscarinic antagonist 5/19). One patient was on long-term low dose oral corticosteroids (Prednisone dose 5 mg) for treatment of rheumatoid arthritis.

Post-bronchodilator spirometry after 1 month of treatment showed moderate FAO (mean ± SD z-score: FEV_1_–2.05 ± 0.75, FVC -0.61 ± 0.95, FEV_1_/FVC -2.46 ± 0.90). After 2 months of treatment FOT indices were abnormal: R_5_ (median (IQR) z-score: 2.7(1.8–3.2)) and X_5_ (z-score: − 3.9(-7.3 - -2.0)). Spirometry did not change between enrolment and after 2 months of treatment, however R_5_ worsened. Eighteen patients performed lung elastic recoil measurements (median (IQR) z-score: K 1.57(− 1.08–3.43) and B/A -1.18(− 1.65--0.02)). Increased compliance was demonstrated in 9/18 patients (K z-score ≥ 1.64) and loss of elastic recoil in 5/18 (B/A% z-score ≤ − 1.64).

Eighteen patients performed bronchoscopy and BAL neutrophil and eosinophil cell counts were obtained in 10 patients (mean ± SD: neutrophils 9.1 ± 18.1% and eosinophils 1.9 ± 1.6%). No patients had evidence of neutrophilic airway inflammation whilst 4/10 patients had eosinophilic airway inflammation. BAL cytokines were obtained in 17 patients and results are shown in Fig. 1. BMI, spirometry, FOT and elastic recoil indices did not correlate with BAL neutrophil or eosinophil count and inflammatory cytokines (Fig. 2). Occasionally, statistically significant correlations were observed however these were the result of a single outlier.

**Fig. 1:**
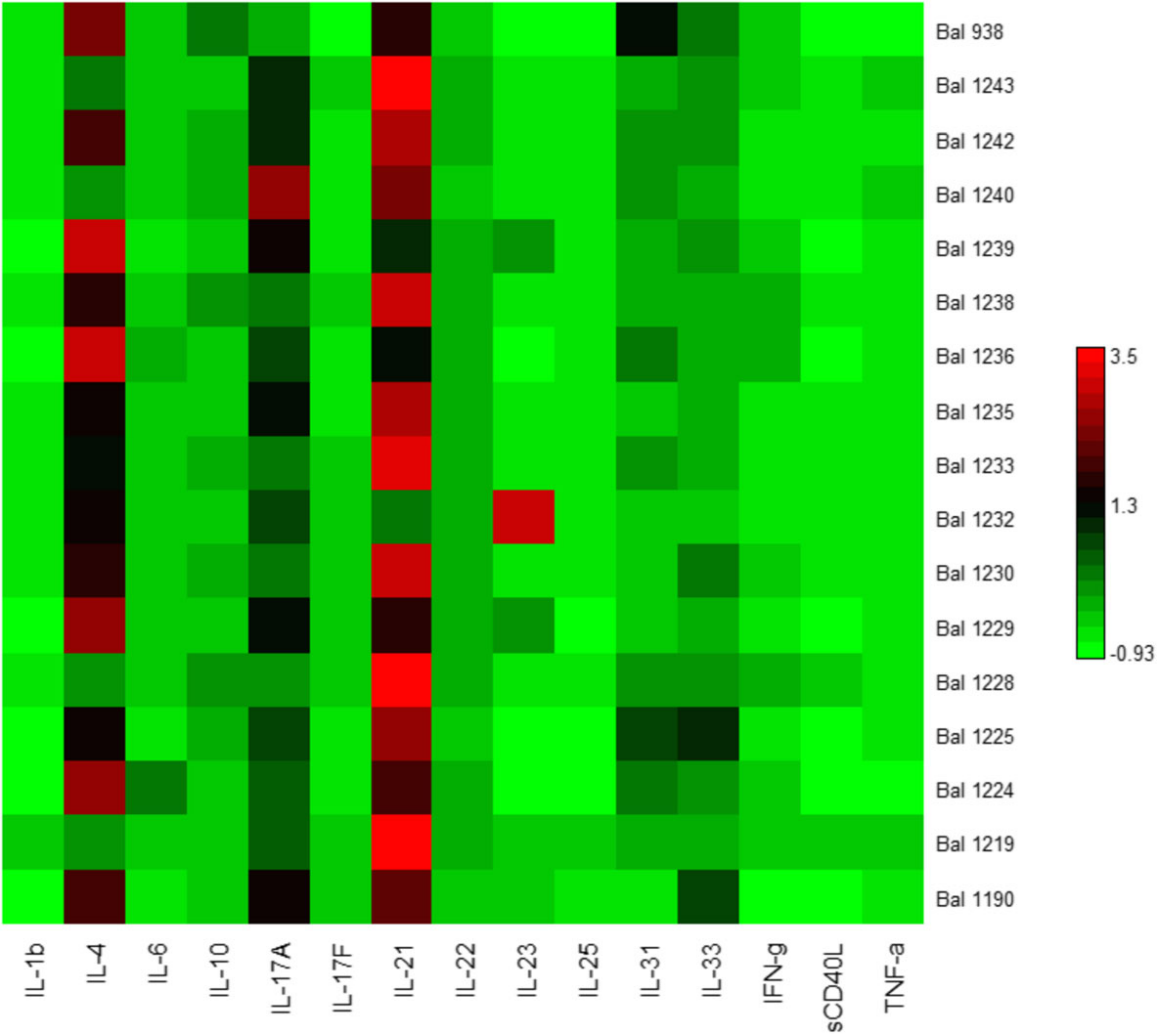
Cytokine levels measured in bronchoalveolar lavage fluid from each patient. Each row represents a patient and each column represents different cytokines. Red indicates highest levels and bright green lowest levels. IL = interleukin, IFN-g = interferon gamma, sCD40L = soluble CD40 ligand, TNF-a = tissue necrosis factor alpha

**Fig. 2:**
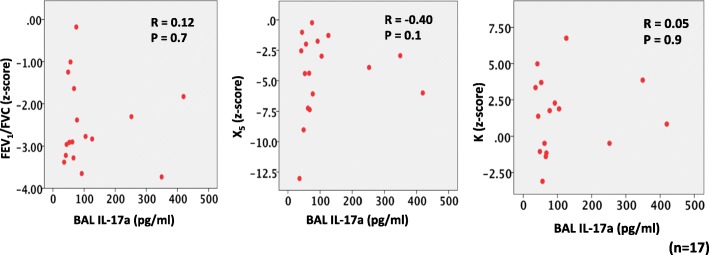
Univariate relationships between lung function measurements and BAL IL-17a. No significant correlations were demonstrated and similar findings were seen with other cytokines. FEV_1_/FVC=forced expiratory volume/forced vital capacity, K=reflects lung compliance, X_5_=reactance at 5Hz, BAL=bronchoalveolar lavage, IL=interleukin

Variable levels of neutrophils, eosinophils and cytokines were detected in this small cohort however there was a disconnect between measures of airway inflammation and lung function. The lack of a relationship in this cohort suggests persisting airway inflammation does not affect lung function following ICS treatment. However, the effect of previous inflammation and inflammation over time on lung function and FAO development remains unknown. A standardized period and dose of ICS treatment was used to minimize potential confounders however most patients were not steroid naïve thus the study treatment may have had minimal additional effect on the inflammatory profile. This is supported by the absence of any significant change in spirometry from enrolment and after the two-month study period. Adherence to study treatment was assessed after 1 month and at the two-month mark to ensure non-adherence did not play a role.

Somewhat surprisingly and in contrast to previous studies [11], this older cohort did not demonstrate neutrophilic airway inflammation although small subject numbers are a limiting factor. Furthermore, neutrophil activation was not measured and may have been increased due to inhibition of neutrophil apoptosis by inhaled corticosteroids. Despite treatment with high-dose ICS/LABA, eosinophilic inflammation persisted in a few patients and cytokines were still detectable, suggesting a steroid unresponsive inflammatory pathway. The fact that FAO develops despite treatment suggests inhaled corticosteroids may have minimal effect on airway remodeling in older people with asthma. Instead FAO in this cohort may predominantly be due to other mechanisms such as the loss of elastic recoil observed in this study, which in turn may occur as a result of lung tissue changes (i.e. lung remodeling) [2]. Lung tissue changes could be due to proteolytic enzymes disrupting lung parenchyma-terminal bronchiole attachments [12]. Inflammation in the lung tissue cannot be ignored, however our study lacks the ability to assess this. Less invasive tests such as a computer tomography (CT) scan to assess for possible lung tissue changes like emphysema [13] was also not done. A recent study demonstrated micro-emphysema, only on microscopic examination of post-mortem asthmatic lungs, which was not evident on CT imaging [2], therefore inclusion of CT imaging may not have be adequate to demonstrate lung tissue changes in our study.

## Conclusion

In summary, this exploratory cross-sectional study has shown a lack of relationship between persistent airway inflammation and lung function impairment following a short period of maximal ICS treatment. However, other cellular mechanisms, lung tissue inflammation and the potential longitudinal effect of inflammation over time in the development of FAO in asthma warrant further investigation.

## Abbreviations

ACQ: Asthma control questionnaire
B/A%: Reflects lung elastic recoil
BAL: Bronchoalveolar lavage
FAO: Fixed airflow obstruction
FEV_1_: Forced expiratory volume in 1 s
FOT: Forced oscillation technique
FVC: Forced vial capacity
ICS: Inhaled corticosteroid
IFN-γ: Interferon- γ
IL: Interleukin
K: Reflects lung compliance
LABA: Long acting-beta-agonist
P-V: Pressure-volume
R_5_: Resistance at 5 Hz
scD40L: Soluble cD40 ligand
TNF-α: Tissue necrosis factor-α
X_5_: Reactance at 5 Hz
